# Vascular cognitive impairment

**DOI:** 10.1590/1980-57642016dn11-040001

**Published:** 2017

**Authors:** Mônica Sanches Yassuda, Lea Tenenholz Grinberg

As invited editors, we are honored to present this Dementia e Neuropsychologia edition which focuses on Vascular Cognitive Impairment (VCI). VCI is an extremely important concept in geriatric and neurologic care. In a broad sense, VCI refers to cognitive deficits associated with vascular disease. It is well known that vascular disease is the second-most-common cause of dementia, after Alzheimer's disease, and an important contributor to mixed dementias. Nonetheless, it is the most preventable and treatable form of cognitive impairment. Thus, it is of great importance to discuss VCI risk factors, diagnosis and treatment strategies.

Identifying VCI may be challenging. It includes diagnosing vascular disease, its severity, the possible brain areas affected, and presence of concomitant risk factors. Frequently, the major challenge is judging to what degree vascular disease can explain the observed cognitive impairment (partially or totally). This clinical judgement can be particularly difficult when VCI is associated with small vessel disease and when the time between disease onset and observation of cognitive deficits is long.

In the present issue, some articles investigated the contribution of neuroimaging and CSF biomarkers to the identification of VCI. Others examined the association of risk factors for VCI, such as hypertension, diabetes and obesity, with cognitive performance. In addition, some of the published articles explored ideal cognitive markers for VCI and one reported the effects of cognitive interventions for elderly individuals with vascular dementia, among other relevant topics.

On closing this editorial section, we highlight that VCI is an extremely important diagnosis, with serious treatment implications. Cognitive deficits associated with vascular causes may be halted and ensuing dementia might be preventable. Therefore, more knowledge is needed regarding risk factors for VCI, ideal cognitive markers and biomarker parameters for its early identification, including neuroimaging. In this regard, the present edition of D&NP contributes significantly to the field.

**Mônica Sanches Yassuda Lea Tenenholz Grinberg**

